# Sheep (*Ovis aries*) training protocol for voluntary awake and unrestrained structural brain MRI acquisitions

**DOI:** 10.3758/s13428-024-02449-6

**Published:** 2024-06-21

**Authors:** Camille Pluchot, Hans Adriaensen, Céline Parias, Didier Dubreuil, Cécile Arnould, Elodie Chaillou, Scott A. Love

**Affiliations:** 1 INRAE, CNRS, Université de Tours, PRC, 37380 Nouzilly, France; 2 Unité Expérimentale de Physiologie Animale de l’Orfrasière, INRAE Centre Val de Loire, 37380 Nouzilly, France

**Keywords:** Ovine, Magnetic resonance imaging, Animal training, Positive reinforcement, Structural images

## Abstract

Magnetic resonance imaging (MRI) is a non-invasive technique that requires the participant to be completely motionless. To date, MRI in awake and unrestrained animals has only been achieved with humans and dogs. For other species, alternative techniques such as anesthesia, restraint and/or sedation have been necessary. Anatomical and functional MRI studies with sheep have only been conducted under general anesthesia. This ensures the absence of movement and allows relatively long MRI experiments but it removes the non-invasive nature of the MRI technique (i.e., IV injections, intubation). Anesthesia can also be detrimental to health, disrupt neurovascular coupling, and does not permit the study of higher-level cognition. Here, we present a proof-of-concept that sheep can be trained to perform a series of tasks, enabling them to voluntarily participate in MRI sessions without anesthesia or restraint. We describe a step-by-step training protocol based on positive reinforcement (food and praise) that could be used as a basis for future neuroimaging research in sheep. This protocol details the two successive phases required for sheep to successfully achieve MRI acquisitions of their brain. By providing structural brain MRI images from six out of ten sheep, we demonstrate the feasibility of our training protocol. This innovative training protocol paves the way for the possibility of conducting animal welfare-friendly functional MRI studies with sheep to investigate ovine cognition.

## Introduction

Structural and functional MRI acquisitions require the individual to be completely immobile to prevent motion artifacts and obtain accurate images. Several strategies have been developed to limit motion during MRI acquisitions. For example, MRI can be performed under general anesthesia (e.g., Aksenov et al., 2015), without anesthesia but with restraint (e.g., Stefanacci et al., 1998), using sedatives (e.g., Behroozi et al., 2018) or without anesthesia or restraint (e.g., Andics et al., 2014).

For most species, MRI is conducted under general anesthesia (Aksenov et al., 2015; Lee et al., 2015; Sadagopan et al., 2015; Simchick et al., 2019; Zhou et al., 2016). This effective method ensures the absence of movement, allows relatively long MRI experiments and can be considered less stressful than for an awake, restrained animal. However, anesthesia can be detrimental to the health of the individual (DeLay, 2016) and modifies brain activity by disrupting neurovascular coupling (Aksenov et al., 2015; Gao et al., 2017). In addition, the use of general anesthesia is not compatible with the study of higher cognitive processes.

To overcome the negative effects of anesthesia, alternative methods to acquire MRI images without anesthesia have been developed. MRI studies have been carried out in awake but restrained animals such as non-human primates (e.g., Hung et al., 2015; Premereur et al., 2018; Stefanacci et al., 1998), birds (e.g., Behroozi et al., 2020), rodents (Tsurugizawa et al., 2012), pigs (Fang et al., 2006), and rabbits (Weiss et al., 2022). In this method, participants are often restrained by their heads with a surgically implanted device that is fixed to the experimental setup and/or enclosed in a restricted space. To limit the potential stress provoked by these constraints, the animals are often habituated to them prior to the actual experiment (Hung et al., 2015; Tsurugizawa et al., 2012).

In recent years, a new method has emerged to perform completely awake, unrestrained brain imaging in dogs (without any medication i.e., anesthesia or sedation). The first acquisitions were successfully performed in the 2010s with fully awake, unrestrained dogs (Andics et al., 2014; Berns et al., 2012; Huber & Lamm, 2017; Jia et al., 2014; for review, see Thompkins et al., 2016). Importantly, this method refined the procedure of conducting MRI with dogs by removing the potentially negative aspects of general anesthesia. Arguably, because the animal can choose to stop at any time, this method also further minimizes stress compared to an animal habituated to being restrained during the acquisition. A detailed description of the steps required to implement this method with dogs was provided by Berns (2013), Strassberg and colleagues (2019), and Karl and colleagues (2020).

To our knowledge, structural and functional brain MRI studies with sheep have only been conducted under general anesthesia (e.g., Barrière et al., 2019; Just et al., 2021; Lee et al., 2015; Love et al., 2022; Nitzsche et al., 2015; Pieri et al., 2019; Schmidt et al., 2012). However, sheep are widely used as an experimental model and contribute notably in neuroscience research (Banstola & Reynolds, 2022; Murray & Mitchell, 2022). In vivo methods such as electroencephalograms (EEG), electromyography (EMG), and functional near-infrared spectroscopy (fNIRS) have been used with fully awake sheep, without any anesthesia (Chincarini et al., 2020; Nicol et al., 2016; Perentos et al., 2016). EEG and electromyography (EMG) were used to detect rumination and eating (Nicol et al., 2016) and fNIRS to assess the cerebral activity of freely moving sheep under different environmental conditions (Chincarini et al., 2020).

Sheep are capable of learning a wide range of tasks involving various degrees of complexity. For example, they can learn to discriminate between images of familiar conspecific faces displaying different emotional states of neutral or negative valence (Bellegarde et al., 2017). They are also able to discriminate “learned-familiar” faces from an unfamiliar one (Knolle et al., 2017). Using the cup task and the tube task, Duffrene and colleagues (2022) showed that sheep can solve inferential conditions based on deductive reasoning. Most studies of sheep cognition require a series of successive phases to successfully achieve the task. In general, sheep are first familiarized with the experimenters to facilitate handling. Then they are habituated to the experimental equipment before being trained on the actual task to be achieved. Finally, the sheep perform the experiment they have been trained for. The duration of the training varies from a few days to several weeks, depending on the complexity of the task, the animal’s experience, and potentially other factors. It is also possible that some participants do not successfully complete the entire task during an experiment. For example, only three out of ten sheep met all the criteria for the tube task (Duffrene et al., 2022); 13 out of 23 learned the complete operant task of Greiveldinger and colleagues (2009); 16 out of 40 learned the task of discriminating faces of familiar conspecifics displaying different emotional states of neutral or negative valence (Bellegarde et al., 2017). However, these studies show that sheep can be trained to perform a wide range of tasks. Thus, sheep may be good candidates for learning the behaviors required to successfully complete brain MRI acquisitions voluntarily, without anesthesia.

In this article, we present a proof-of-concept that sheep can be trained to perform a series of tasks, enabling them to achieve structural MRI acquisitions of their brains without anesthesia or restraint. We provide a step-by-step training protocol based on positive reinforcement (food and praise) that could be used as a basis for future research in sheep. This protocol details the successive phases required for sheep to successfully complete a structural MRI acquisition of their brain.

## Methods

### Ethics

The study was approved by the local ethical committee for animal experimentation (CEEA VdL, Tours, France, authorization #26807-2020080314491255v6). All methods were performed in accordance with the European directive 2010/63/EU for animal protection and welfare used for scientific purposes.

### Animals

**Fig. 1:**
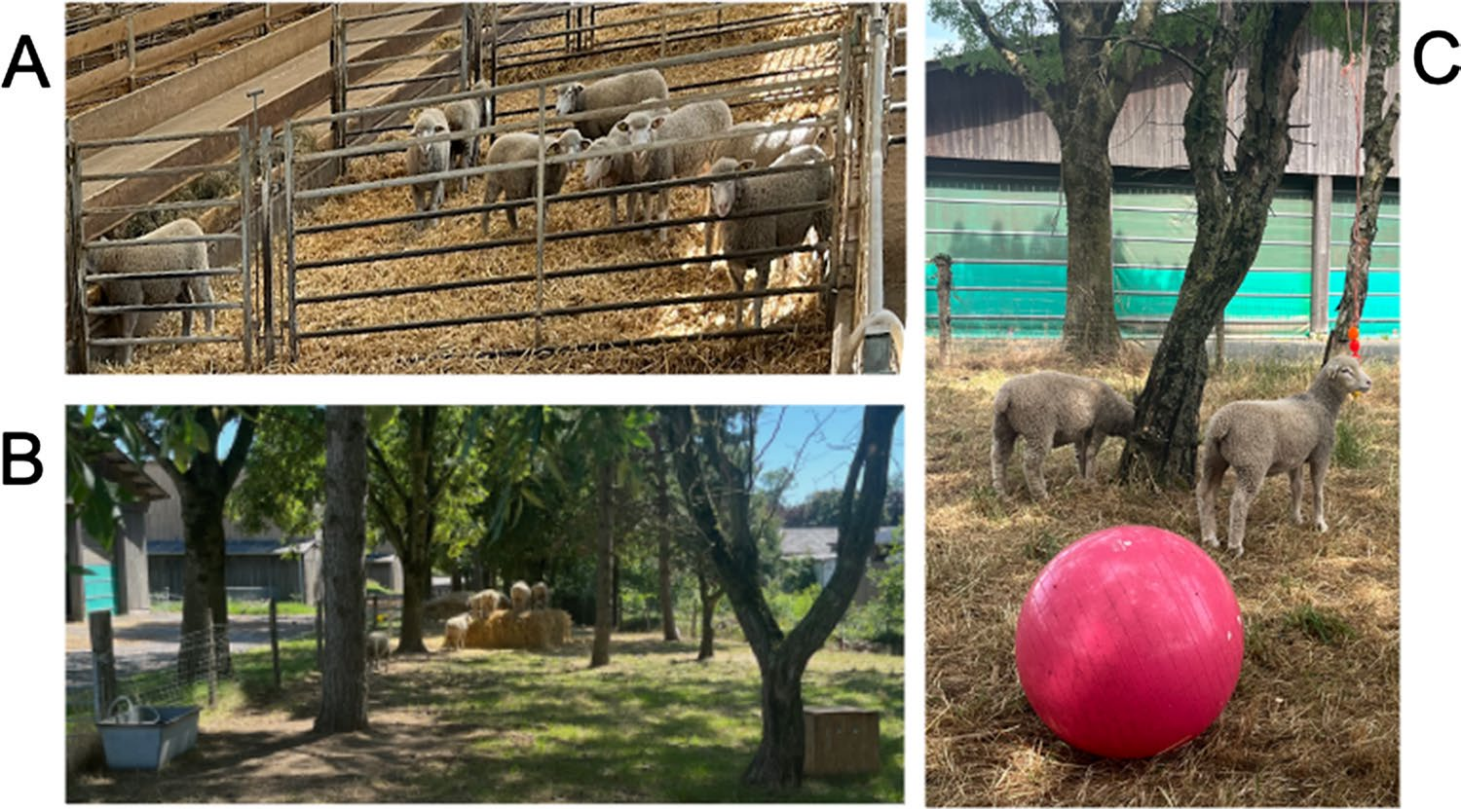
Housing of the ten sheep of the MRI group depending on the weather. **A** Inside the sheepfold; **B, C** Outside in a field with an enriched environment

This study was conducted with Ile-de-France sheep born in early March 2022 at the “Unité Expérimentale de Physiologie Animale de l’Orfrasière” (UEPAO, INRAE Val de Loire, France; 10.15454/1.5573896321728955E12). Within the conventional breeding system of the UEPAO, lambs stay with their mother until weaning at around 80 days of age. However, due to excessive numbers in a litter, non-maternal ewes or health problems, some lambs need to be separated from their mother and artificially reared, at which point they were placed in a single pen equipped with an automatic milk feeder, straw, and hay. After this separation, the lambs were trained to drink formula milk from rubber teats attached to the milk feeder (Förster Technik® TAP5-EZ2), first every 3 h and then once a day until they were capable of feeding themselves in this way. From 2 weeks after birth, lambs had access to feed pellets. However, regular consumption of the pellets only began around the time of weaning (approximately 45 days after birth). At weaning, the milk was withdrawn and the lambs were fed with a solid daily food diet of hay and pellets. From the flock of artificially reared lambs, five males and five females were selected to participate in the training protocol – the MRI group. The five males were castrated before puberty (≈ 4 months after birth) to maintain a stable mixed sex group and to avoid aggressive behaviors towards the trainers. The MRI group was housed either in a sheepfold or outside in a field depending on the season/weather (Fig. 1A, B). In their home pen (20.7 m^2^) of the sheepfold they had *ad libitum* access to water and mineralized salt, while daily quantities of pellets and hay were distributed. The field (2020 m^2^) was equipped for an *ad libitum* access to water and mineralized salt. The sheep could graze in the field but a supplement of hay and pellets was also distributed. Toys (e.g., balls, plastic cubes hung in trees, traffic cones) and bales of straw were placed in the field to enrich the environment (Fig. 1B, C).

### Trainers

Four main trainers (two men: SAL, DD; two women: CaP, CeP) implemented the training protocol. None had any previous experience in animal training. Punctually, eight other people helped with the training. During a training session at least two trainers were present. This was necessary for safety and because the training was physically demanding. All trainers received safety training before being permitted to enter the MRI room.

### Training philosophy

Our training approach was based on positive reinforcement, a positive human–animal relationship and voluntary cooperation of the animal. We chose to begin the training protocol with lambs reared without their mother almost directly from birth based on the fact that it is easier to create a positive trainer–lamb relationship in this context (Boivin et al., 2001; Guesdon et al., 2016). For positive reinforcement, we used food reward, verbal encouragement, cuddling, and stroking. After successful performance of a requested behavior, the lambs were rewarded with one or a combination of these reinforcers depending on the individual's preference. Food reward was also used to lure the sheep into conducting specific behaviors. In general, our strategy was to adapt our training protocol to the performance of each individual. Because of the differences in motivation between each individual of the MRI group, we did not impose a specific delay for the learning of the required behaviors. Moreover, it was possible that the lambs did not cooperate during a training session, in which case they were not forced to work and training was stopped until the next session. We ensured that the lambs were free to move, never tethered to the MRI table, and could leave the training session at any time. Sheep are a very gregarious species that will display extreme signs of distress if isolated from the flock. For this reason, we initially planned to always train one sheep in the presence of at least one other. However, unlike in the case of dogs (Andics et al., 2014), our attempts to train with more than one sheep at a time proved problematic. The sheep tended to interact with each other. Therefore, we finally chose to train and acquire data with one individual at a time. To do so, we habituated them to not feel isolated from the group when they were in the presence of a trainer.

### MRI group selection

For 2 weeks after the lambs were separated from their mother, two trainers (CaP, SAL) spent approximately 2 h with them in the single pen twice a day, 7 days a week. The main aims of this phase were to familiarize lambs to humans and to select the ten individuals of the MRI group. Initially, the trainers sat almost passively in the single pen waiting for the lambs to interact with them. Over the next few days, the trainers increased from passively to actively interacting by placing a hand on their back, stroking their back, scratching under their neck or stomach, etc. When the majority of lambs were clearly comfortable with the presence of the trainers, a variety of objects were introduced into the single pen: baby toys, foam, and a ramp. These objects enriched the environment and some were specifically chosen to begin introducing the behaviors to be learned during the two phases of the training protocol. The trainers encouraged the lambs to walk onto the ramp and also placed the foam on, or gently wrapped it around, their head. Based on the positive interactions expressed by individual lambs toward the trainers and their ease with the objects introduced into the single pen, ten lambs were selected and moved to the home pen (Fig. 1). These ten lambs (five males: Léonard, Ted, Tony, Joe, and Jackson; five females: Brook, Maggie, Robin, Lily, and Barnita) then participated in the training protocol.

### Training protocol

After selecting the ten lambs of the MRI group, the training protocol was divided into two main phases: (1) Learning MRI acquisition-related behaviors and; (2) Training in the real MRI room. Each phase consisted of successive steps performed in the same order for each lamb (Table 1) but not necessarily at the same time. We predicted that the noise produced by the MRI scanner could seriously hinder the chance that sheep would be able to stay motionless inside the scanner. For this reason, they were habituated to the sounds of the MRI scanner throughout the two phases of the protocol. Initially, MRI sounds were occasionally played via a Bluetooth speaker (Bang & Olufsen Beosound A1 2nd generation) for about 5 min in the home pen to the entire MRI group. The volume of the sound was gradually increased over time from 60 to 85 dB (measured at the position of the head using a sound level meter, CESVA, SC-2c). Then, it was often the case that when an individual began to achieve a particular training step, we would have them perform it also in the presence of the MRI sounds. Based on our observation that the lambs performed better in the morning than in the afternoon, we chose to conduct the majority of our training sessions in the morning. In general, a session lasted approximately 10–20 min per individual.

**Table 1:** Behaviors of the step-by-step training protocol

Training phase	Step	Training criterion
Learning MRI behaviors		
(Phase 1)	1	Climb a ramp to reach the mock MRI table
	2	Lie down on the mock MRI table
	3	Stay still on the moving mock MRI table
	4	Place head in the mock RF head coil
	5	Wait motionless with the head inside the mock coil (at least 5 min)
+	Habituation to the scanner noise (from 60 to 85 dB over sessions)	
Real MRI room		
(Phase 2)	1	Climb the ramp to reach the patient MRI table and lie down on the table
	2	Lie still while the table rises and lowers
	3	Headphones on the ears
	4	Place head in the real coil with foam to wedge their head
	5	Move forward to the center of the scanner bore
	6	Stay motionless inside the scanner bore with MRI sounds
+	Habituation to the scanner noise in the real MRI environment	

### Learning MRI acquisition-related behaviors (phase 1)

**Fig. 2:**
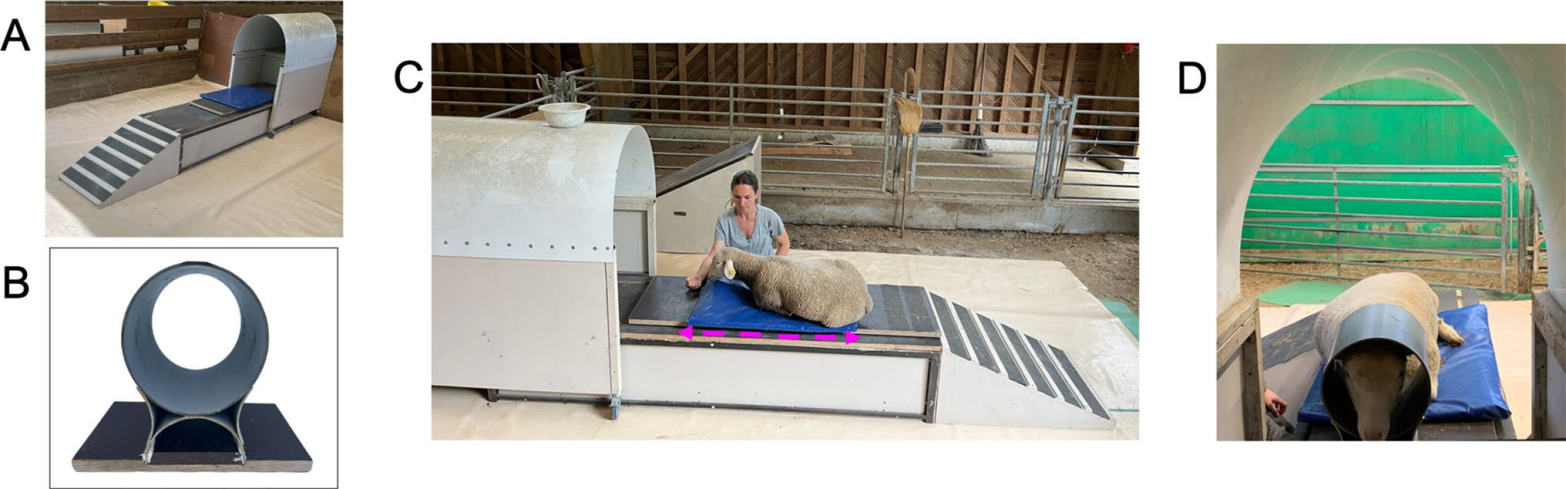
Learning MRI acquisition-related behaviors. **A** The mock MRI scanner composed of a ramp, a fixed base on which a mock patient bed sits and a mock bore; **B** The mock RF head coil; **C** Ted lying down on the moveable mock MRI table (*dashed pink arrow*), on a comfortable blue mat, in front of the mock MRI bore; **D** Léonard waiting motionless with his head inside the mock RF head coil

Phase 1 training sessions took place in the training pen (20.7 m^2^), which contained custom-made mock MRI acquisition equipment (mock MRI scanner with ramp, Fig. 2A, mock radio frequency (RF) head coil, Fig. 2B). The home pen and the training pen were adjacent and connected by a gate. During the first week of training, the ten lambs were trained together and could move freely between the home and training pens. They were then trained in groups of four and were no longer allowed free access to the two pens. After 1 month of training, the ten lambs were each trained alone.

During phase 1, the lambs were trained in MRI-related behaviors following five successive steps: climbing the ramp, lying down on the mock MRI table, staying still while the mock MRI table moved back and forth, being equipped with the mock RF head coil and waiting motionless for several minutes (Table 1, Fig. 2, C-D). Phase 1 lasted approximately 5 months, during which the lambs were trained 4–5 times a week.

**Fig. 3:**
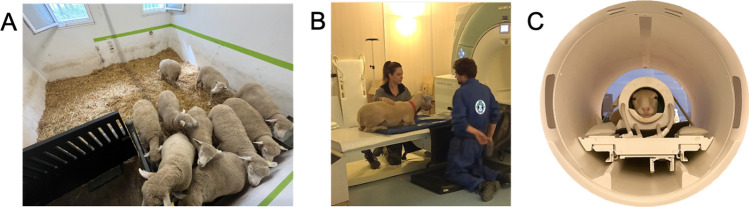
Training in the MRI room. **A** The MRI group in the waiting pen at the MRI platform; **B** Brook lying down on the patient bed; **C** Ted waiting motionless and listening to the sounds of the MRI scanner. His head is inside the sheep RF coil and positioned at the center of the bore

In detail, for this phase of the training protocol, the mock MRI scanner and the mock RF coil were custom-made by the technical staff of the laboratory. The mock MRI scanner was built based on the dimensions of the real scanner and made of metal, wood, and plastic. It consisted of a fixed base (length = 234 cm x width = 63 cm × height = 44 cm), a movable patient bed (length = 160 cm × width = 50 cm) and a mock bore (length = 141 cm × width = 73 cm × height = 142 cm). The mock patient bed had wheels that were inside rails, enabling it to be pushed in and out of the mock bore. The spherical mock RF head coil (diameter: 24 cm; Fig. 2B) was created using PVC plastic to mimic our actual spherical 24-channel sheep’s head coil (diameter: 20 cm; RAPID Biomedical GmbH, Rimpar, Germany, 1H Phased Array for Sheep Brain P-H24LE-030-01808; Fig. 3C). A ramp with a slip-resistant surface (length = 140 cm × width = 63 cm × height = 51 cm, slope = 34%) was built to allow lambs to walk up and down onto the patient table. The height of the ramp was equivalent to the real MRI patient table in its lowest position.

The lambs were trained using French verbal signals to indicate a desired behavior: “patte” for learning to bend their front legs over their knees, “couché” for learning to bend their back legs and lie down (step 2 in Table 1), and “pose” for learning to stay motionless. The sheep learned to stay in the lying down position while table movements were initiated manually back and forth (step 3 in Table 1), to move their head in the right position into the mock MRI bore (step 4 in Table 1). Foam padding was placed between the head and mock RF coil to minimize head motion. At this time of the training protocol, the lambs were trained to wait (step 5 in Table 1). The interval between posing the head inside the coil and the reward was gradually increased (a few seconds at the beginning to 5–10 min at the end of this step). During this step, the lambs must be as motionless as possible to express the behavior required during real MRI sessions.

### Training in the MRI room (phase 2)

This phase of the training took place within the MRI room at the imaging platform PIXANIM (Phénotypage par Imagerie in et ex vivo de l'Animal à la Molécule, 10.17180/CQ4D-DW26). As the MRI was located approximately 500 m from the animal facilities, the sheep learned to walk between their home pen and the imaging platform where they were housed together in a waiting pen (surface: 15 m^2^, Fig. 3A) before and after a training session. At the end of each training session, the sheep walked back to their home pen. The aim of this training phase was for the sheep to implement the learned MRI acquisition-related behaviors in the real MRI environment. The main differences between the mock and the real MRI environments are the noise, the temperature, the light, the table lifting up and down via a controlled step motor, the height of the table, and meeting new people working at the platform. Moreover, the sheep had to acquire new skills such as accepting to wear headphones, which were essential for protecting their hearing. They also had to remain motionless in the MRI bore with all the equipment required for acquisition (headphones, thicker foam around the head, etc.). This phase included six steps (real MRI room phase; Table 1) during which the sheep were trained 2–5 times a week for about 3 months.

**Fig. 4:**
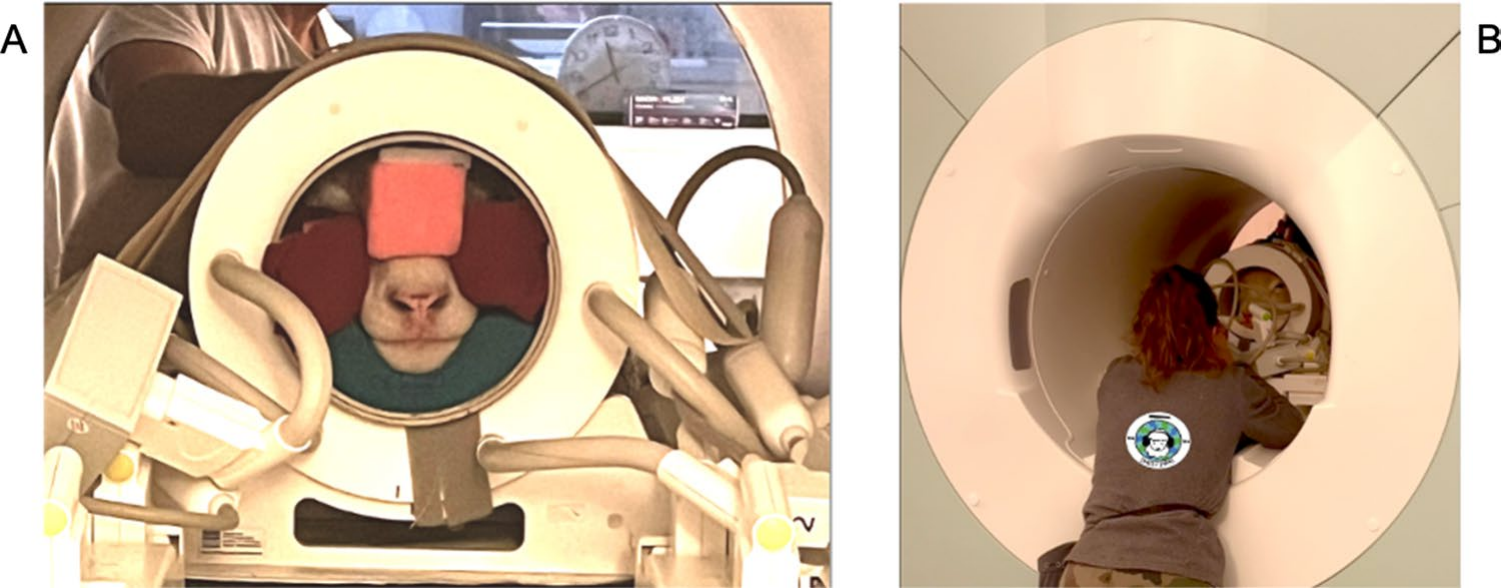
Sheep performing brain MRI acquisition. **A** The head position in the sheep RF coil. The head is “blocked” with foam pieces adapted to each individual. A trainer is at the front of the bore with a hand placed on the sheep’s back; **B** The other trainer sat at the back of the bore, facing the sheep, to maintain visual contact

The first training step was to express previously learned behaviors in the novel environment of the MRI room: climb up the ramp and lay down on the patient MRI table (Fig. 3B). To facilitate this step, the ramp used during phase 1 was placed alongside the patient table in its lowest position. A plastic-covered foam mat was placed on the patient table to provide greater comfort, prevent damage, and make it easier to clean the table. The second step required the sheep to lie still while the table automatically rose and lowered. Once the sheep were lying on the table in the raised position, we put headphones (MR Confon, Magdeburg, Germany) on their ears (step 3 in Table 1). To maintain the headphones in place, a headband was created with muslin tights. The next step (step 4 in Table 1) was to use the real 24-channel head coil. For safety, the coil was attached to the table with straps (Fig. 3C), but the sheep were free to place and remove their head from it at any time without injury to them or damage to the coil. Once the sheep's head was well positioned in the center of the coil, foam was placed around the snout to minimize motion (Fig. 4A). These pieces of foam have been specifically created to fit the shape of a sheep's snout. The number and thickness of the foam pieces used depended on both the sheep's ability to keep their head motionless in the coil and their head size. The sheep was then automatically moved forward to the center of the scanner bore (step 5 in Table 1). Once the sheep was placed in the center of the bore, they were asked to stay motionless (step 6 in Table 1). During step 6, the time between the moment the sheep was moved to the center of the bore and the reward delivery was gradually increased (within and between sessions). The rate of the interval increase was entirely dependent on the sheep. It is important to note the position of trainers during this step. One trainer directly faced the sheep from the back of the bore while, at least, one other was at the front of the bore with a hand placed on the sheep’s back. Once sheep were capable of lying motionless in the center of the bore for several minutes (2–4 min), MRI sounds were played (volume from 60 to 85 dB). Once step 6 was completed, the sheep had successfully learned all the behaviors required to perform MRI acquisitions.

### Brain MRI acquisitions

During an awake MRI acquisition session, sheep were required to implement the MRI acquisition-related behaviors that they had learned. Our proof-of-concept awake sheep MRI session took approximately 5 min. The MRI was conducted using a 3T Siemens Magnetom® Verio scanner (maximum gradient amplitude of 45 mT/m). Data acquisition included an initial localizer scan (33 s) followed by a T1-weighted structural image of the whole brain (2 min, 36 s) using a 3D magnetization-prepared rapid gradient echo (MPRAGE) sequence (TR = 2200 ms, TE = 2.64 ms, flip angle = 12°, 448 × 448 matrix and, FOV = 500 mm with a slice thickness of 1.12 mm resulting in an isotropic 1.12 × 1.12 × 1.12 mm^3^ voxel size). We chose a T1-weighted image as our proof-of-concept because it is a standard sequence for structural brain imaging. However, alternative and potentially faster sequences are also available for structural imaging (e.g., T2-weighted; Berns et al., 2012, 2013). The sheep were in a head-first, prone position (Fig. 4). Slices were oriented sagittally to the sheep’s brain with the phase-encoding direction ventro-dorsally.

## Results

### Group selection

From the artificially reared lambs, the ten selected were those that displayed the most positive interactions (strokes, food reward) with the trainers and the most ease with the MRI-related objects. During the MRI group selection, all ten lambs expressed a spontaneous preference for the trainers. Lambs approached the trainers and to a lesser extent other humans as soon as they were near the pen but they only sought contact with trainers. The gradual increase in the number of people working with them (four main trainers and eight other people) during the training protocol enabled all lambs to spontaneously approach unfamiliar people. Two months after the beginning of familiarization with the trainers, the ten lambs showed this ability to generalize to all humans. At 2–3 weeks of age (mid-March 2022), all five females and one male (Joe) accepted to have a piece of foam held around their head. The four other lambs (Jackson, Léonard, Ted, and Tony) didn’t easily accept the foam around their head. However, as they interacted well with the trainers and the other MRI-related objects they were chosen to be part of the MRI group.

### Learning MRI acquisition-related behaviors (phase 1)

**Fig. 5:**
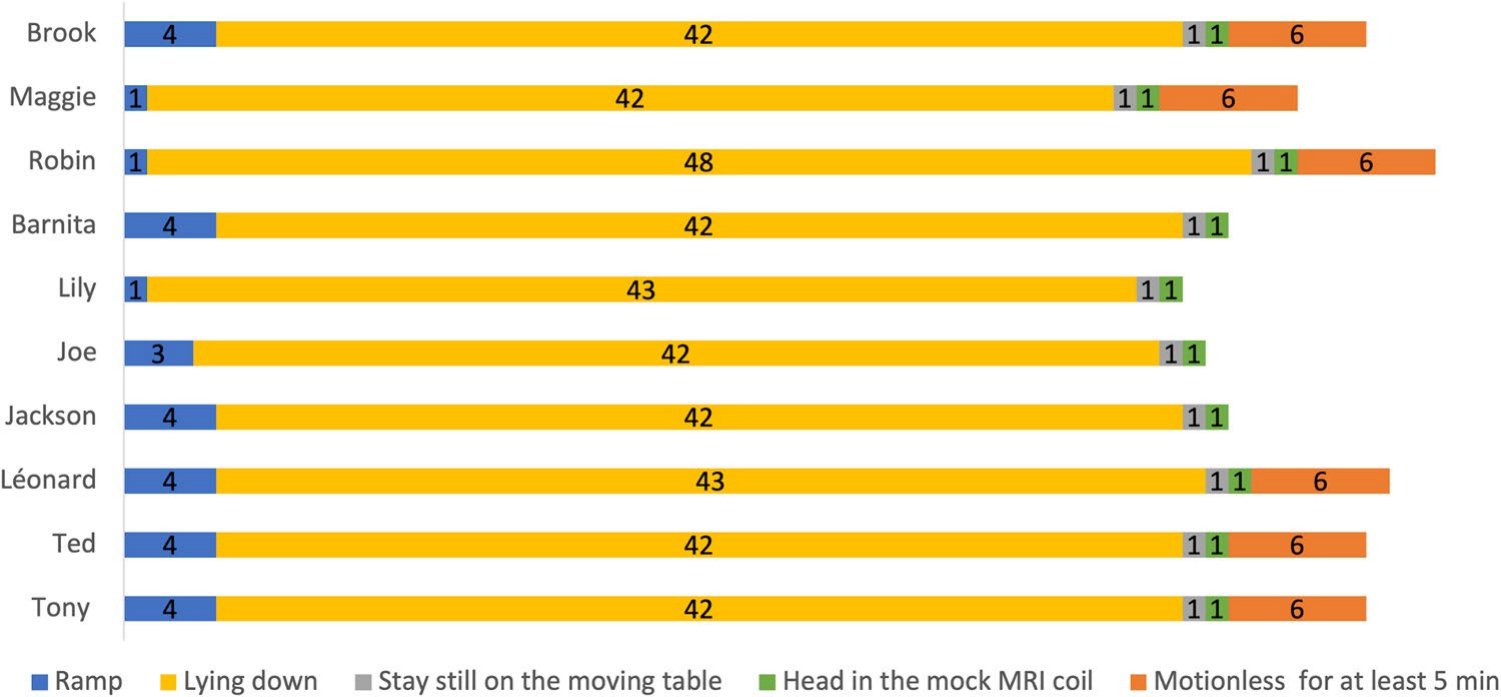
Number of training sessions for each individual (Brook, Maggie, Robin, Barnita, Lily, Joe, Jackson, Léonard, Ted, Tony) and for each step to be achieved during phase 1. Note that the absence of the *orange line* for Barnita, Lily, Joe and Jackson indicates that this step was not successfully completed

After selecting the MRI group, the mock MRI environment was set up and training sessions began in the training pen. The lambs came spontaneously into the training pen and quickly interacted with the MRI-related equipment. Throughout the different steps of phase 1, progress differed between the individuals (Fig. 5). From the first training session with the ramp, Maggie, Robin, and Lily walked up and down the ramp to receive a reward. It took three training sessions for Joe and four for Brook, Barnita, Jackson, Léonard, Ted, and Tony to achieve this step. In four training sessions, all ten lambs in the MRI group were capable of walking up and down the ramp to reach the mock MRI table.

Figure 5 shows that the “Lying down” behavior was the most time-consuming step of phase 1. During this step, which lasted approximately 2 months, the lambs learned to lie down on the table by first bending their front legs to follow a food lure, then their hind legs. The ten lambs needed between 42 and 48 training sessions to learn how to lie down on the mock MRI table by themselves, following the verbal signals corresponding to the requested behavior. Once the “Lying down” behavior was learned, forward and backward movements of the mock MRI table were initiated. No habituation was required, as from the first training session for this step, the lambs stayed still on the table while it was in motion. The mock RF head coil was introduced during the next training sessions. Once again, no habituation was required for this step. The lambs could be lured to place their head in the mock RF coil from the first training session with this equipment.

The last step of this phase of the training protocol consisted of teaching the lambs to wait motionless with their head in the mock RF coil for at least 5 min. Brook, Maggie, Robin, Léonard, Ted, and Tony successfully achieved this step after six training sessions. The four other lambs of the MRI group (Barnita, Lily, Joe, Jackson) are not yet capable of waiting motionless long enough with their head in the mock RF coil. However, we decided to begin training in the real MRI environment with all sheep, as it was evident that they were obedient and calm and that they would not harm themselves or the MRI equipment.

### Training in the MRI room (phase 2)

**Fig. 6:**
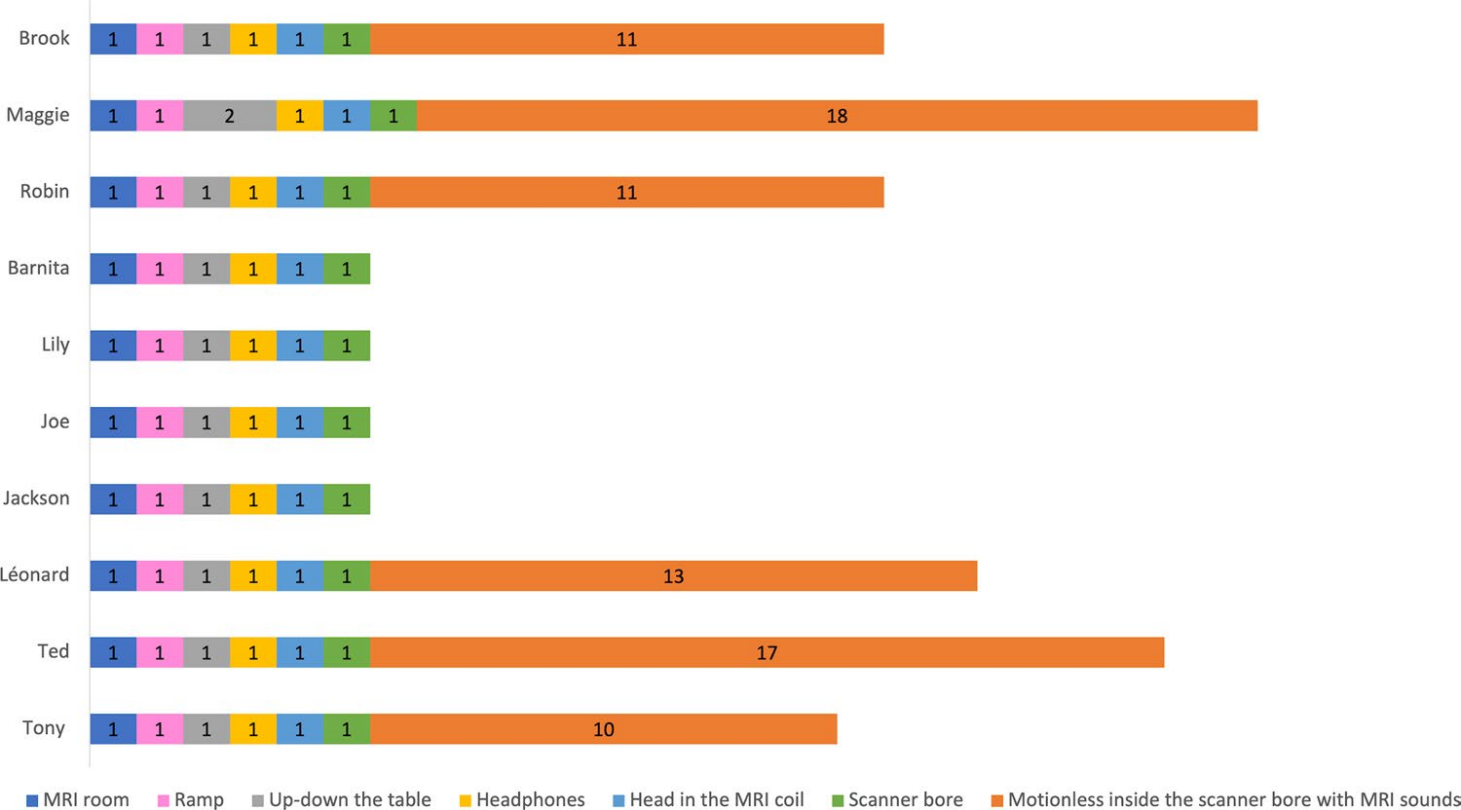
Number of training sessions for each individual (Brook, Maggie, Robin, Barnita, Lily, Joe, Jackson, Léonard, Ted, Tony) and for each step to be achieved during phase 2. Note that the absence of the *orange line* for Barnita, Lily, Joe and Jackson indicates that this step was not successfully completed

For each training session in the MRI room, the ten sheep had to walk from their home pen to the imaging platform. Trips were conducted with the whole MRI group. The ten sheep always walked voluntarily between the imaging platform and their home pen. The first training session in the MRI room took place from August 26, 2022, with Maggie, Robin, and Ted (approximately 5 months old) to October 26, 2022, with Barnita. From their first training session, each of the sheep entered the MRI room, walked up the ramp and lay down on the MRI table (Fig. 6). They did not show any stress reaction (no vocalization, no defecation, etc.). Except for Maggie, they all accepted that the patient table was elevated to its highest position. Maggie accepted that the table was raised during the second training session in the MRI room. These results suggest that the sheep generalized the MRI-related behaviors learned during phase 1 to the real MRI environment of phase 2.

MRI acquisitions are extremely noisy, reaching sound pressure levels of up to 130 dB. Thus, headphones are placed over the sheep's ears to protect them from this noise (30 dB of passive gradient noise suppression). During the first training session involving headphones, none of the ten sheep displayed any negative reaction to wearing either the headphones or the headband wrapped around their head to keep them in place.

The next step consisted of placing the head in the RF coil with foam between the coil and the head to wedge them in and minimize head movements. Once again, no habituation was required for this step. The sheep could be lured into placing their head inside the coil and accepted to have foam placed around their heads from the first attempt. A single training session was then sufficient to know that the sheep would remain lying down, with their head inside the coil until the table reached the center of the scanner bore.

The final step in this training protocol was to teach the sheep to stay motionless inside the scanner bore for several minutes, with their head inside the RF coil and listening to the MRI sounds. However, Barnita, Lily, Joe, and Jackson have not yet reached the final step of phase 2 of the training protocol. They are still learning to stay motionless inside the scanner bore long enough for an acquisition. The other six sheep (three females and three males) in the MRI group successfully achieved this step. Tony learned to stay motionless in the scanner bore for several minutes, with his head inside the RF coil, while listening to the MRI sounds in ten training sessions, and Robin and Brook in 11 training sessions, Léonard in 13 training sessions, Ted in 17 training sessions, and Maggie in 18 training sessions.

**Fig. 7:**
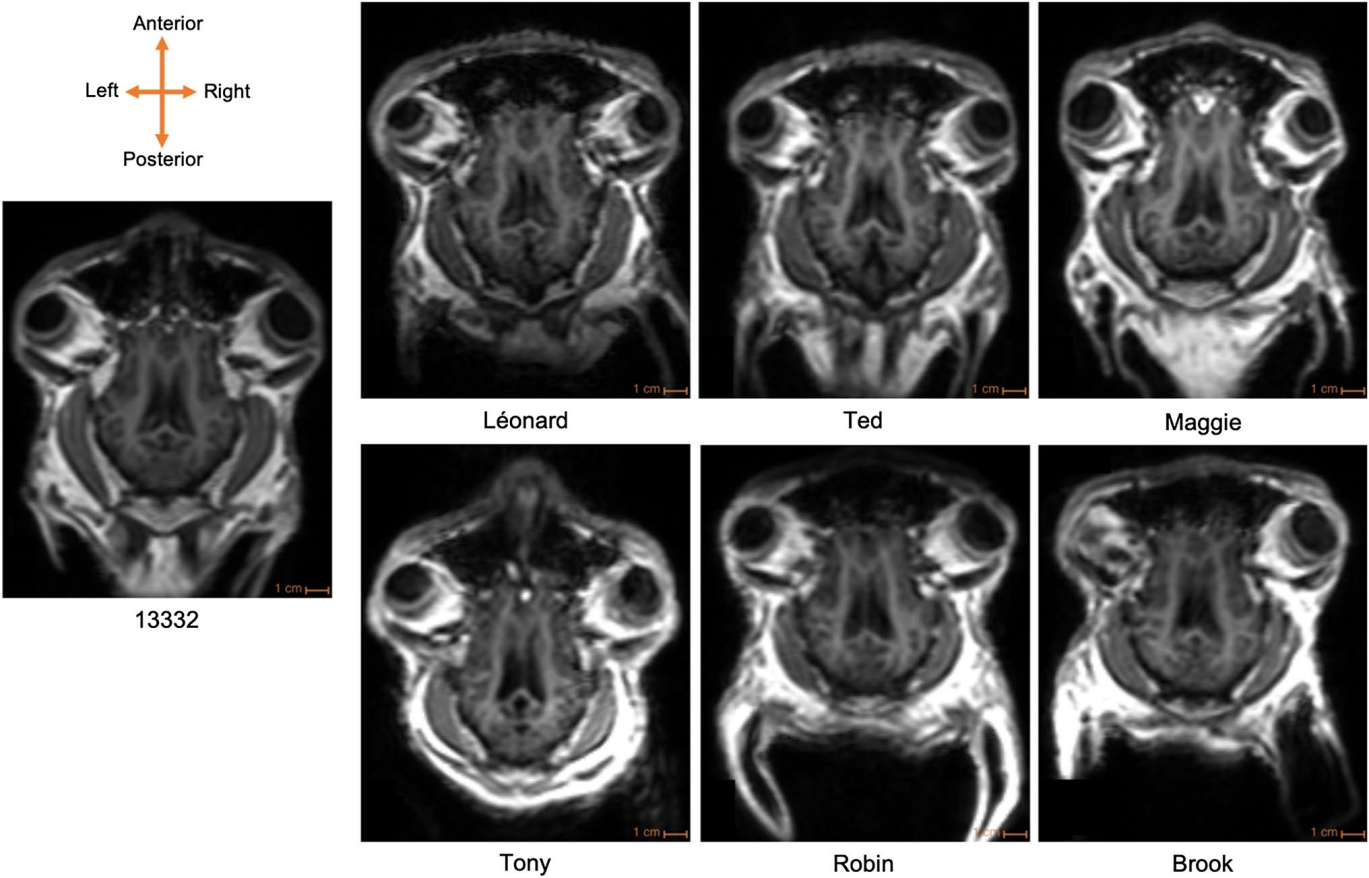
Comparison of T1-weighted structural acquisition (axial slices) with anesthetized ewe (13332) and awake-unrestrained sheep (Léonard, Maggie, Brook, Robin, Ted, Tony)

Success in this final step for six of the sheep was marked by the acquisition of a T1-weighted structural image without the need for anesthesia or restraint after almost 9 months of training (Fig. 7, data accessible at: 10.5281/zenodo.10044453). A video showing a successful acquisition can be viewed here, 10.5281/zenodo.10213970.

It is important to note that the lambs were habituated to the sounds of the MRI scanner first in their home pen with the entire group, then alone during phases 1 and 2 of the training protocol. No specific reaction (withdrawal, attempt to flee, vocalizations, ear movements, etc.) was noticed when the recorded MRI sounds were played in the different locations (i.e., home pen, training pen, or MRI room), or to the sounds of an actual acquisition.

## Discussion

This study proposes a step-by-step training protocol, based on positive reinforcement, to enable sheep to perform MRI acquisitions over several minutes without anesthesia or restraint. One of the aims of this work was to refine the procedure of conducting MRI with sheep by removing the potentially negative health effects of general anesthesia and to preserve neurovascular coupling, which is disrupted by anesthetic agents (Aksenov et al., 2015). To date, T1-weighted structural images have been acquired from six out of the ten sheep in the MRI group. The resulting images are comparable to those obtained in sheep under general anesthesia, demonstrating the feasibility of acquiring MRI brain images without the use of anesthetic agents or physical restraint of the individual. Awake sheep can remain as motionless as sheep under general anesthesia in a noisy and spatially enclosed MRI environment. Using this training protocol, anesthesia-related side effects are avoided and cognitive processes could be investigated in future fMRI studies with awake and unrestrained sheep.

This protocol was an ideal approach for teaching sheep the behaviors required to successfully perform structural MRI acquisitions of their brain. Despite the time-consuming nature of the training protocol, overall it was relatively straightforward to implement. As with dogs, we used a step-by-step training protocol involving training with a mock MRI scanner to learn the MRI acquisition-related behaviors, then implementing these behaviors during training sessions in the real MRI room (Berns, 2013; Karl et al., 2020; Strassberg et al., 2019). Despite the differences in temperature, brightness, and noise between the MRI room and the training pen, the sheep easily transferred from the training pen to the MRI room (i.e., a single training session for steps 1–5 in phase 2) and showed no negative reaction. In this way, the sheep generalized the acquisition behaviors they had learned during the phase 1 training pen sessions. Therefore, the training sessions in the real MRI environment really began directly at the step, “staying motionless inside the scanner bore for several minutes, with their head inside the RF coil”. However, four sheep have not yet succeeded in this final step of phase 2, which we highlight as a critical step in this training protocol.

MRI acquisitions require the individual to be completely immobile to prevent motion artifacts and obtain accurate images. However, it is demanding for both human and non-human animals alike to remain motionless in a closed, noisy environment for several minutes (Enders et al., 2011; King et al., 2005; Lukins et al., 1997). Teaching sheep to hold their heads motionless inside the enclosed sheep RF coil was a critical step in this training protocol. Six sheep in the MRI group successfully achieved this last step, enabling them to perform T1-weighted acquisitions. The four other sheep are still not yet able to stay motionless long enough with their head inside the RF coil to perform an MRI acquisition. We believe that there are at least three possible reasons for this. First, as it became clear that these four sheep were progressing slower than the six others, we began to spend less time training with them and more time with the six other sheep. Second, perhaps our choice of training technique was less appropriate for these four sheep and did not enable them to understand the instructions well enough to successfully complete this step of the protocol. Recently, we have started to use the "clicker training" technique (Skinner, 1951) with them and we have seen improved progress in their ability to stay motionless. Third, it is also possible that they, and a certain proportion of sheep in general, will never be capable of staying motionless long enough to acquire MRI data. However, we feel that this is the least likely possibility and that with enough time and the correct technique the vast majority of sheep will be capable of completing an MRI acquisition.

We also identified that the “lying down” behavior was a critical step for all the sheep in this protocol. Initially, it was complicated to find a technique that could lay the sheep down. After approximately 20 training sessions, we discovered the appropriate technique (described in the Method section). Then after another approximately 20 sessions, all ten sheep successfully completed this step. Therefore, repeating this protocol with a new group of sheep would take less time since we already have a well-developed technique to lay the sheep down (a video of this technique can be viewed at 10.5281/zenodo.10213970).

Our training protocol is based on positive reinforcement, with a social or a food reward for each correctly performed behavior. Initially, we tried different options of food reward (carrot, apple, beet biscuits, and pellets) to motivate the performance of the behavior. Lambs showed no real interest in apples, carrots (used in Duffrene et al., 2022 with adults) or beet biscuits but only for pellets. These pellets are those used in the daily diet and are the most suitable food reward for Ile-de-France sheep in a livestock context. However, before weaning, the lambs were not motivated by solid food, as they were fed exclusively with a milk diet at that stage of development. Therefore, social rewards played an important role in the beginning of the training protocol in particular. The sheep were very receptive to praise and strokes from the first training sessions as they had experienced such positive interactions from birth during the familiarization with trainers. We believe that building this positive trainer–lamb relationship was important for the success of this training protocol. This is based on the fact that, unlike other sheep, they easily explored new environments and objects and accepted training without conspecifics but only in the presence of trainers. These subjective observations suggest that trainers may be considered as congeners for the sheep. An open question in this regard is whether it is necessary or not to build this positive trainer–lamb relationship from birth.

In conclusion, we present a proof-of-concept that sheep can be trained to voluntarily perform brain MRI acquisitions without anesthesia and without restraint. Our training protocol is perfectly suited to teaching sheep the behaviors required for an MRI acquisition. It provides an animal welfare-friendly protocol limiting any form of physical restraint by never tethering sheep to the MRI table and allowing them to leave at any time during a session. The motivation of both sheep and trainers at every step of this reward-based training ensured the success of the protocol. Arguably, our criteria for success were too stringent, as other MRI sequences that require less time could still be of interest experimentally. For example, echo-planar imaging of the BOLD response to short blocks (e.g., 8–16 s per condition) of sensory stimulation could help to uncover the neural mechanisms underlying a multitude of cognitive processes. We are currently acquiring such data with our MRI group. By acquiring T1-weighted structural images with sheep, a new species has joined the era of awake, unrestrained neuroimaging.

## Data Availability

The data generated during the current study are available in the Zenodo repository, 10.5281/zenodo.10044453. Two supporting videos are also available: one illustrates our technique to lie sheep down, while the other shows the acquisition of a T1-weighted image from an awake and unrestrained sheep 10.5281/zenodo.10213970.

## Declarations

### Open Practices Statement

The data acquired in this work are available at 10.5281/zenodo.10044453. As this work was not hypothesis driven it was not preregistered.
